# Congenital Cervical Hemivertebrae and Block Vertebrae in a 43-Year-Old Male

**DOI:** 10.7759/cureus.18812

**Published:** 2021-10-16

**Authors:** Margarida M Freitas, Luisa C Ventura

**Affiliations:** 1 Physical and Rehabilitation Medicine, Hospital Garcia de Orta, Almada, PRT

**Keywords:** spine instability, klippel-feil syndrome, congenital spine deformity, block vertebra, cervical hemivertebra

## Abstract

Congenital deformities of the spine are a consequence of anomalous vertebral development in the embryo and may be identified at birth or remain unnoticed until adulthood. Minor bony malformations of all types occur in up to 12% of the general population and are usually not apparent. In contrast, congenital spinal malformations that result in progressive spinal deformity are relatively rare. Klippel-Feil syndrome is a complex heterogeneous entity that results in cervical vertebral fusion and sometimes can occur associated to hemivertebra.

We present a case of a 43-year-old male who presented to the emergency department after a fall. The patient had severe cervicalgia and generalized loss of active movement and sensation on his limbs. On physical examination, the patient presented reduced cervical range of motion and tetraplegia. Cervical magnetic resonance imaging revealed complete atlanto-occipital assimilation, left C3 hemivertebra with partial fusion in the right lateral portion of C2 and C4. Finally, there was a C5-C6 fusion. Surgical cervical stabilization was attempted but the procedure was aborted due to lack of space for placing the anterior plate safely. During hospitalization, the patient wore a cervical collar and started a rehabilitation program including muscle strengthening, balance, and gait training. The rehabilitation treatment led to a favorable clinical evolution. At discharge, the patient maintained a slight deficit of strength in his left upper and lower extremities, but he was functionally autonomous and was able to walk with a walker.

## Introduction

Congenital abnormalities of the cervical spine are a broad spectrum of bony deformities, ranging from asymptomatic anatomical variants to multiorgan-system syndromic anomalies. Some spine deformities may result in chronic pain, biomechanical instability, or spine compression [1]. Congenital changes in the cervical spine include hemivertebrae, block vertebrae, atlantal anomalies, and occipitalization of the atlas [1].

Hemivertebrae result from a lack of formation of one side of a vertebral body. This abnormality is caused by failure of formation or segmentation of somites during osteogenesis [2]. The estimated incidence is around 0.3 per 1000 live births [3]. The half segment may be fused to one of the adjacent vertebrae, above or below. The hemivertebrae can be classified according to the region that is ossified. If the vertebra has only the posterior vertebral components, it is a dorsal hemivertebra and results in kyphosis. Lateral hemivertebrae classically result in scoliosis and ventral hemivertebrae result in lordosis [4]. Once a hemivertebra is identified, careful assessment should be done to identify any coexistent skeletal, cardiac, genitourinary, and gastrointestinal anomalies [5].

Block vertebrae of the cervical spine are a rare congenital deformity. Some authors consider block vertebrae an accidental radiological finding unrelated to any disease, while others hypothesize that they might cause secondary degenerative changes and mobility disturbances of adjacent segments [6]. Fusion of multiple cervical vertebral bodies may be a sign of Klippel-Feil syndrome or vertebral defects, anal atresia, cardiac defects, tracheoesophageal fistula, renal anomalies, and limb abnormalities (VACTERL) association [4]. Kippel-Feil syndrome may increase the risk of a transient neurologic deficit or spinal cord injury (SCI) after minor trauma. This can be explained by the altered mechanical force transfer caused by the fused segments and the excessive mobility of the non-fused ones [7].

Tetraplegia is defined as the impairment or loss of motor and/or sensory function in the cervical segments of the spinal cord due to damage of neural elements within the spinal canal [8]. The severity of tetraplegia can range from transient to permanent injury. Neuropraxia is the less severe form of cervical SCI and is defined as a transient loss of motor or sensory function that can last from less than 15 minutes to 48 hours. The mechanism of injury is usually hyperflexion, hyperextension, or direct axial load of the cervical spine [9].

The American Spinal Cord Injury Association (ASIA) created a classification of the type and severity of the lesion - the ASIA impairment scale (AIS). This scale assigns each patient a letter, from A to E, which is associated with the severity of the injury. In brief, A means a complete spinal lesion, B represents sensory incomplete, C indicates a motor incomplete lesion, D stands for motor incomplete injury, and E means that sensation and motor function are graded as normal in all segments in a patient with prior deficits. The muscle function grading includes 10 paired myotomes, which are tested bilaterally, C5 to T1 and L2 to S1. A six-point scale is used for scoring, ranging from zero (total paralysis) to five (normal muscle strength and function) [8].

This study aimed to emphasize the importance of multisystemic screening when a vertebral anomaly is found. Also, we aim to highlight that some deformities can go unnoticed until adulthood, when they can have severe complications. This case report is interesting due to its rarity, severity, and impressive clinical images.

## Case presentation

A 43-year-old male with spina bifida occulta presented to the emergency department after a traumatic brain injury followed by a fall, without loss of consciousness. The traumatic brain injury was caused by an accident at work, involving storage boxes falling over the patient. He presented severe cervicalgia, anterior cervical flexion, and generalized loss of active movement and sensation. On physical examination, the patient presented reduced cervical range of motion, short neck, low posterior hairline, apparent cervical kyphoscoliosis, and tetraplegia. Cervical kyphoscoliosis had never been investigated or treated previously. Around two hours after the accident, the patient presented hypoalgesia below T2 level. Muscle strength grading according to the AIS was 3/5 in left finger flexors, 4-/5 in left elbow extensors and finger abductors, 4-/5 in left hip flexors and knee extensors, with no other neurological deficits. According to the American Spinal Injury Association Impairment Scale, the patient had a C7 AIS D lesion [8]. This classification means that C7 is the neurological level of injury, and it refers to the most caudal segment of the spinal cord with normal sensory and antigravity motor function on both sides of the body [8]. In this case, the patient had a D lesion, with motor function preserved at the most caudal sacral segments (tested by voluntary anal contraction) and had some sparing of motor function in his upper and lower extremities. To be considered a D lesion, the patient must have a muscle grade equal or superior to 3/5 in at least half of key muscles below the neurological level of injury (C7).

**Figure 1:**
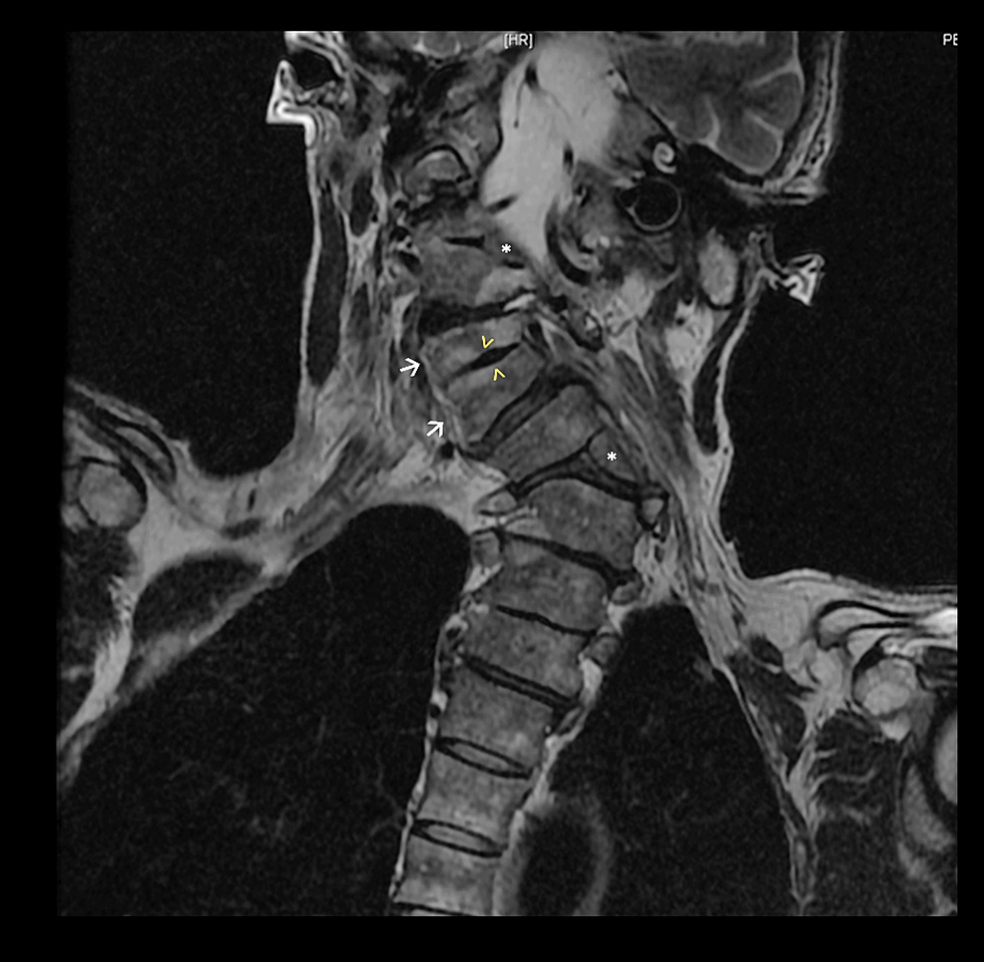
Cervical MRI, coronal plane, T2-weighted image Cervical MRI showed no signs of fracture. Complete atlanto-occipital assimilation is observed. There is a left C3 hemivertebra (upper asterisk), not segmented, associated with partial fusion in the right lateral portion of C2 and C4. There is a C5-C6 block vertebra (white arrows). The intervertebral disc between these two vertebrae is degenerated (arrowheads) and partially absent, and there is an incomplete bone fusion. There is another hemivertebra, presumably T1 (lower asterisk).

**Figure 2:**
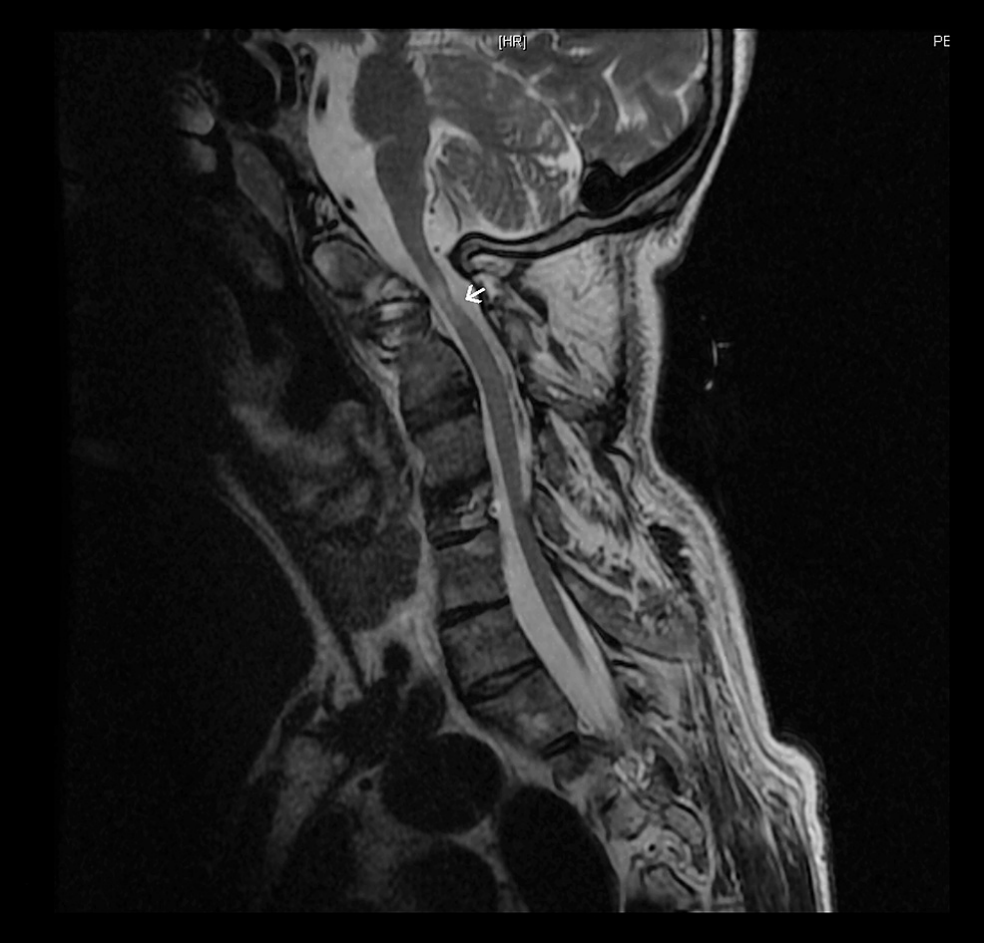
Cervical MRI, sagittal plane, T2-weighted image Spinal canal stenosis is visible at C1-C2 level, associated with spinal cord hyperintensity (arrow).

Regarding radiological findings, the cranial magnetic resonance imaging (MRI) was normal. The patient had both thoracic and lumbar abnormalities with incomplete T1 vertebra and spina bifida occulta (L4 posterior vertebral defect). Cervical magnetic resonance imaging revealed important additional findings, which are described in Figures 1, 2.

Surgical cervical stabilization was attempted but the procedure was aborted due to lack of space for placing the anterior plate safely. During the 22 days of hospitalization, the patient wore a cervical collar and started a rehabilitation program including muscle strengthening, balance, and gait training. At discharge, he maintained a slight deficit of strength in his left upper and lower extremities but was able to walk with a walker. At discharge, the patient maintained a classification of C7 AIS D lesion. Rehabilitation treatment continued in an outpatient clinic and included 12 weeks of physiotherapy and occupational therapy sessions three times a week.

The presence of spina bifida, scoliosis, hemivertebrae, and block vertebrae led to the following differential diagnosis: anatomical variant, Klippel-Feil syndrome (KFS), Wildervanck syndrome, VACTERL association, and Aicardi syndrome. The patient did not have deafness or strabismus, which excluded Wildervanck syndrome [4]. Imaging examination did not find tracheoesophageal, kidney, or cardiac anomalies. The absence of cleft palate, limb abnormalities, cardiac defects, or anal atresia excluded VACTERL association [4]. Aicardi syndrome was excluded because the cranial MRI did not reveal agenesis of the corpus callosum and physical examination did not show any characteristics of Klinefelter syndrome. Aicardi syndrome only affects women or males with 47, XXY chromosome constitution [10]. The most likely diagnoses were Klippel-Feil syndrome (KFS) or anatomical variant. During hospitalization, the patient was asked to carefully read and sign an informed consent for publication and use of clinical images. Researchers ensured data confidentiality.

## Discussion

KFS is a rare congenital condition with an estimated incidence of 1:40,000, and it is characterized by the abnormal fusion of cervical vertebrae [11]. Clinical research using cervical computed tomography scans reported a higher incidence of KFS, up to 1.2%, suggesting there may be a large number of underdiagnosed patients [12]. KFS may be caused by different gene mutations, including growth differentiation factor 6 (GDF6), growth differentiation factor 3 (GDF3), and mesenchyme homeobox 1 (MEOX1) [4].

The classic clinical presentation includes a triad of short neck, low posterior hairline, and limitation of neck mobility [11], but fewer than 50% of the patients present with the classic triad [13]. Decreased range of neck movement is the most common clinical finding. KFS can occur in association with Goldenhar syndrome, anomalies of the extremities, scoliosis, spina bifida, torticollis, facial nerve paralysis, Chiari I malformation, or Duane’s contracture of the lateral rectus muscle [11,14]. Also, between 30% and 60% of the patients with KFS have genito-urinary problems, including unilateral renal agenesis, malrotation of the kidney, ectopic kidney, and ureteral duplication [15].

Classically, Klippel-Feil syndrome is classified into three types: type I - massive fusion of cervical and upper thoracic vertebrae, type II - fusion of only one or two pairs of cervical vertebrae, and type III - cervical fusion in combination with lower thoracic or lumbar fusions [15]. A more recent three types of following radiological classification of congenital spinal fusion by Samartzis et al. is being used for KFS: type I - single cervical, type II - multiple non-contiguous segments, and type III - multiple contiguous cervical fusions [16]. Regarding prognosis and complications, KFS is associated with early degenerative changes throughout the cervical spine, spinal canal stenosis, fracture, spondylosis, disc degeneration, and disc herniation [16].

Concerning KFS treatment, it must be patient-oriented and tailored. There are no specific guidelines or contraindications. Treatment options include analgesic medication, physical therapy, patient and family education, and cervical stabilization surgery [17]. Physical therapy consists of gentle soft tissue massage, aerobic and postural exercises [18]. Spinal manipulation should be avoided due to the risk of cervical instability and neurological damage. For transient quadriplegia, soft cervical collars and bracing are often suggested. Patient and family education are essential for fall prevention, home modification, and repositioning of overhead objects. Non-contact sports and aquatic exercises should be favored over contact sports [18]. Surgical treatment of KFS is indicated in patients with cervical instability, persistent pain, or acute neurologic deficit. Symptomatic spinal stenosis may require decompression and fusion [18]. We consider this clinical report very relevant to the medical community due to its rarity and impressive MRI images.

## Conclusions

Cervical congenital spine deformities may go unnoticed until adulthood. Even though the signs and symptoms are obvious to the clinical eye, they may not be appreciated by the patient. These deformities are a risk factor for degenerative cervical myelopathy and traumatic spinal cord injury. Early identification of cervical congenital spine deformities and modification of activities may reduce the risk of subsequent trauma and prevent degenerative vertebral disease.

Hypermobility of the upper cervical spine presents a risk for neurologic compromise, whereas hypermobility of the lower cervical spine causes degenerative disc disease. The treatment of KFS is directed toward the specific symptoms and physical findings. Such treatment requires a multidisciplinary team and includes physical therapy, use of cervical collars, analgesics, or vertebral fixation surgery.
